# Docking-based virtual screening of known drugs against murE of Mycobacterium tuberculosis towards repurposing for TB

**DOI:** 10.6026/97320630012368

**Published:** 2016-11-22

**Authors:** Sridharan Brindha, Jagadish Chandrabose Sundaramurthi, Devadasan Velmurugan, Savariar Vincent, John Joel Gnanadoss

**Affiliations:** 1 Loyola College, Nungambakkam, Chennai – 600034, Tamil Nadu, India; 2 National Institute for Research in Tuberculosis (ICMR), Chetpet, Chennai – 600031, Tamil Nadu, India; 3 Centre of Advanced Study in Crystallography and Biophysics, University of Madras, Guindy Campus, Chennai - 600025, Tamil Nadu, India

**Keywords:** Repurposing, Drugs, Tuberculosis, Virtual Screening, Bioinformatics, murE

## Abstract

Repurposing has gained momentum globally and become an alternative avenue for drug discovery because of its better success rate, and reduced cost, time and issues related to safety than the conventional drug discovery process. Several drugs have already been successfully repurposed for other clinical conditions including drug resistant tuberculosis (DR-TB). Though TB can be cured completely with the use of currently available anti-tubercular drugs, emergence of drug resistant strains of Mycobacterium tuberculosis and the huge death toll globally, together necessitate urgently newer and effective drugs for TB. Therefore, we performed virtual screening of 1554 FDA approved drugs against murE, which is essential for peptidoglycan biosynthesis of M. tuberculosis. We used Glide and AutoDock Vina for virtual screening and applied rigid docking algorithm followed by induced fit docking algorithm in order to enhance the quality of the docking prediction and to prioritize drugs for repurposing. We found 17 drugs binding strongly with murE and three of them, namely, lymecycline, acarbose and desmopressin were consistently present within top 10 ranks by both Glide and AutoDock Vina in the induced fit docking algorithm, which strongly indicates that these three drugs are potential candidates for further studies towards repurposing for TB.

## Background

New drugs for the effective treatment of tuberculosis are greatly needed now than ever due to the increasing trend in the emergence of drug resistant strains of Mycobacterium tuberculosis (M. tuberculosis), especially in India, China and Russian Federation [1]. Poor success rate of the treatment of drug resistant tuberculosis (DR-TB) than drug sensitive TB alarmingly indicates for newer and effective drugs urgently for the successful treatment and control of TB, globally [1]. It has been reported that the current drug discovery pipeline for TB appears to be healthy since a good number of candidate drugs are being evaluated in different phases of clinical and preclinical research [2,3]. However, emergence of resistant mycobacteria even for newer drugs also cannot be ruled out in the future, thus, search for newer and alternative drugs for the treatment of TB should be a continuous process to ensure the sustained control of TB.

The conventional drug discovery process is challenged by several obstacles including expensive budget, lengthy process for discovery of new compounds, and most importantly meagre success rate [4]. Identifying new applications for already FDAapproved drugs for clinical conditions other than intended use is becoming a promising new avenue to pursue for drug discovery, a process defined as repurposing or repositioning [4]. Strategy of repurposing of already approved drugs appears to be a viable approach since it reduces the time spent in establishing pharmacokinetics and safety issues for human use [4]. This approach has been already adopted as a successful strategy in various clinical conditions including, premenstrual dysphoria (fluoxetine, primarily used for depression), multiple myeloma (thalidomide, primarily used for sedation, nausea and insomnia) [4]; the process of repurposing has recently been recognised and well supported by international agencies including National Institute of Health, USA and Medical Research Council, UK for research funding to explore possible repurposing of drugs for various diseases and to understand their mechanisms [5]. Fluoroquinolones, clofazimine, linezolid are few drugs that are successfully repurposed in the treatment of drug resistant tuberculosis [6].

The genome of M. tuberculosis encodes for about 4000 proteins [7]. Therefore, selection of a protein as a drug target is crucial for drug discovery for TB. Extensive research, including bioinformatics based studies has been carried out to identify and prioritize drug targets for TB [8,9]. Essentiality of protein for the pathogen and absence of homolog in eukaryotes are commonly used criteria for drug target selection, which we have used in the present study. The gene murE (Rv2158c) is an essential gene for the Mycobacterium tuberculosis since it encodes for a protein, UDPN- acetylmuramoyl-L-alanyl-D-glutamate--2,6-diaminopimelate ligase that catalyzes a biochemical reaction essential for the biosynthesis of peptidoglycan of M. tuberculosis and it does not have a homolog in eukaryotes [10]. Therefore, murE has been suggested as a promising target for new drug discovery for tuberculosis and thus, its crystal structure has been determined in the presence of the substrate, UDP-MurNAc-l-Ala-d-Glu (UAG) [11]. Based on these evidences, we selected murE of M. tuberculosis as the drug target in the present study and performed virtual screening for 1554 FDA approved drugs using Glide and AutoDock Vina. We employed rigid docking algorithm and subsequently induced fit docking algorithm in order to enhance the quality of the docking prediction and to prioritize drugs for repurposing for TB.

## Methodology

### Preparation of drug target and drug-library

Atomic coordinates of murE was downloaded from Protein Data Bank (PDB ID: 2WTZ) [12] which has been used in previous study for docking based drug discovery [13]. Protein preparation wizard (Schrödinger, USA, 2014) was employed to prepare the target protein file for virtual screening. The structure was prepared for docking by following the standard procedure which includes, addition of hydrogen, assigning bond orders, removal of water molecules, optimization of hydrogen bonds, and energy minimization. The drug library consists of 1554 FDA approved drugs which were obtained from DrugBank [14]. LigPrep (Schrödinger, USA, 2014) was used to prepare the drug-library since it helps to prepare high quality, single, low-energy, 3D structure with correct chiralities, and apply force field for energy minimization for each entry of the chemical structure provided in the drug-library.

### Virtual screening

**Figure 1:**
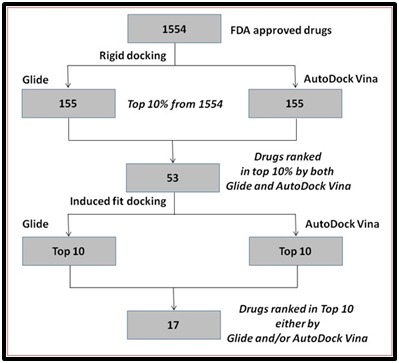
The protocol employed in the prioritization of drugs against murE of M. tuberculosis.

All the 1554 drugs retrieved from DrugBank were screened against murE independently by using two docking methods namely Glide (Schrödinger) and AutoDock Vina (ADV) through PyRx (0.8) [15,16]. In the initial round of screening, rigid docking was employed; for the selected drugs which were present in top 10% in the rigid docking by both Glide and ADV, induced fit docking was performed for further prioritization. Using Glide, extra precision (Glide XP) method was used to generate grid around the active side residues with default settings and Glide score was used to rank the drugs after docking. AutoDock Vina was used as the second method through PyRx software in order to obtain consistent results, and binding energy of the docked complex was used to rank and shortlist drugs. The procedure applied in the present study has been illustrated in Figure 1. Molecular interactions were also analysed for all of the shortlisted drugs with murE. RMSD value of the docked complex and original crystal structure of the target were also calculated. Binding affinity, molecular interactions and RMSD value were collectively used to prioritize drugs as potential candidates for repurposing for tuberculosis.

## Results and Discussion

Virtual screening of 1554 FDA approved drugs against murE of M. tuberculosis was performed using two different molecular docking tools and by employing rigid docking followed by induced fit docking algorithms. At the end of first round of docking (rigid docking) 53 drugs were ranked within top 10% by both Glide and AutoDock Vina. Further, induced fit docking of the 53 shortlisted drugs with murE enabled us to prioritize 17 drugs as they were found within top 10 ranks either by Glide or ADV (Figure 1). A total of 3 drugs, namely lymecycline (rank 3 by Glide and rank 1 by ADV), acarbose (4 and 2) and desmopressin (7 and 3) were ranked consistently within top 10 ranks by both Glide and ADV. RMSD value for each of the docked complex relative to the crystal structure of the murE was calculated and it was observed that all of the 17 shortlisted drugs had value lesser than 1Ao. More details about these 17 prioritized drugs, including binding affinity with murE, RMSD value, and molecular interactions with the target protein, and the primary use of each of the drugs are provided in the Table 1.

**Figure 2:**
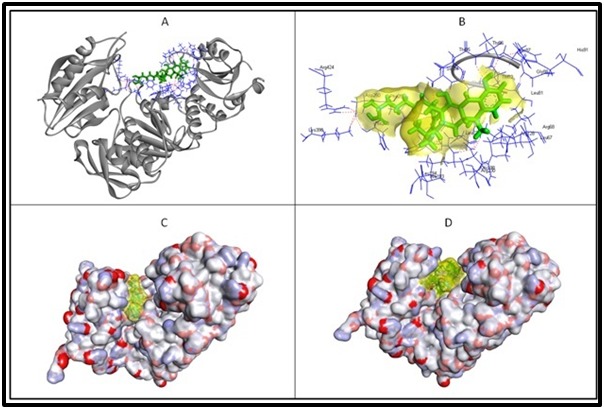
Molecular interactions of murE and three of the prioritized drugs. Figure A displays the target protein in solid ribbon form, gray in colour with interacting amino acids highlighted in line form, blue in colour while the docked lymecycline is displayed in stick form, green in colour. The intermolecular hydrogen bonds are displayed in dotted lines, red in colour. The magnified view of the interactions for better clarity is provided in the Figure B; semi-transparent surface over lymecycline (yellow in colour) and interacting amino acids labelled with three letter code. The effective fitting of the acarbose and desmopressin in the active site groove of the murE is clearly displayed in figure C & D respectively; acarbose and desmopressin is highlighted in semi-transparent surface (yellow in colour) and displayed in stick form.

Based on the crystal structure of the murE, amino acids, T195, S222, R230, E198, L67, S84, Q70, A69, T86, T85 were reported to involve in the molecular interactions with UAG [11,12]. Molecular interactions between shortlisted drugs and murE were analysed and the interacting amino acid residues are provided in the Table 1 and those residues known to be present in active site of the murE are provided in boldface [11,12]. Lymecycline was found to be the top most drug by ADV ranking and rank 3 by Glide scoring. When the molecular interactions were analysed, it was found that lymecycline interact with seven amino acids of murE (Table 1) including, S84, T86, E198 and R230 which are reported to be present in the active site of the murE [11,12]. The RMSD value of the lymecycline and murE docked complex relative to the crystal structure of the murE was found to be 0.922Ao. Acarbose was another drug ranked consistently within top 10 ranks by both ADV (2) and Glide (4) and the RMSD value was calculated to be 0.91Ao. Acarbose was found to interact with 11 amino acids of murE (Table 1); among them, eight residues (S84, S222, R230, T195, T85, T86, Q70 and L67) are present in the active site of the murE [11,12]. Similarly, desmopressin was ranked 3 by ADV and 7 by Glide and the RMSD values were 0.927Ao. Desmopressin was also found to interact with 11 amino acids including eight amino acids from the active site of the murE (S84, R230, T195, T86, T85, L67, A69 and Q70) [11,12]. In an earlier study of murE of M. tubeculosis carried out by Singh et al (2014), most of the amino acids from the active site were demonstrated to interact with docked molecules, thus, such molecules were suggested as potential inhibitors of the enzyme [17]. Consistent ranking by both Glide and AutoDock Vina and by rigid docking as well as induced fit docking, strong molecular interactions with that of amino acids in the active site of the murE and significant RMSD values collectively suggest that these three drugs, lymecycline, acarbose and desmopressin are high-confident drugs for repurposing for TB. Molecular interactions between murE and the three drugs, lymecycline, acarbose and desmopressin have been illustrated in the Figure 2. Among these three drugs, lymecycline is primarily used for the treatment of various other bacterial infections [14], which led us to form a hypothesis that 'lymecycline would affect M. tuberculosis by blocking its peptidoglycan biosynthesis by targeting murE', which needs to be validated experimentally.

Out of the 17 drugs prioritized by multiple rounds of docking, in addition to lymecycline, six more drugs, namely fidaxomicin, indinavir, caspofungin, vancomycin, micafungin and polymyxin B sulfate are also known to be used against bacterial, viral or fungal infections (Table 1) [14]. The mechanism of these drugs, in general, they inhibit essential proteins of the respective pathogens, thus, blocking their life cycle in one way or the other. For example, lymecycline binds to bacterial 30S ribosomal subunit and prevent translation of proteins [14]. Vancomycin prevents gram-positive bacterial cell-wall biosynthesis by inhibiting the incorporation of N-acetylmuramic acid and Nacetylglucosamine peptide subunits into the peptidoglycan matrix [14]. Indinavir is known to inhibit protease of HIV that is required for proteolytic cleavage of the viral polyprotein precursors into the individual functional proteins [14]. Strong binding of these seven drugs (which are primarily used against other pathogens) with murE of M. tuberculosis indicates that they could be potential candidate drugs for repurposing or serve as leads for new drug discovery for tuberculosis.

The prioritized drugs in the present study have come through several rounds of filtering, thus, they are worthy for further investigation by experimental studies. The aim and scope of our present study is to prioritize drugs by in silico virtual screening and suggest them for further studies for repurposing. However, we performed literature search to know whether any of the prioritized drugs are already validated in vitro against tuberculosis. Interestingly, it was observed that totally 4 drugs, namely acarbose, fidaxomicin (Synonyms: lipiarmycin), vancomycin and polymyxin B sulfate have been reported to have anti-mycobacterial activity by other researchers. Caner et al., (2013) demonstrated acarbose to strongly bind to trehalose synthase (treS) which is involved in essential functions such as energy storage, signaling, protein-protection and bacterial cell wall components of M. tuberculosis [18]; thus, Caner et al (2013) reported that acarbose could be used as a competitive inhibitor of treS of M. tuberculosis [18]. Fidaxomicin is also known as lipiarmycin, was demonstrated to have inhibitory activity against multidrug-resistant strains of M. tuberculosis [19]. Recently, vancomycin and tetrahydrolipstatin were reported to show synergistic inhibitory activity on M. bovis BCG and on M. tuberculosis [20]. Polymyxin B (synonymous: polymyxin B sulfate) was reported to show bactericidal activity against M. smegmatis [21]. Indinavir was shortlisted as a potential drug for repurposing for TB by another bioinformatics based study [22]. Since four of the 17 drugs are already reported to have anti-mycobacterial activity in vitro, we are encouraged to suggest these 17 prioritized drugs as potential candidates for further exploration towards repurposing for TB. Additionally, the same fact that four of the prioritized drugs have in vitro anti-mycobacterial activity strongly supports our procedure as a valid one and convinces us that it can be used as a generic protocol for prioritization of drugs for repurposing for other clinical conditions. Our protocol has identified 12 existing drugs as potential candidates for repurposing towards TB for the first time based on virtual screening against murE of M. tuberculosis. We started the present study with 1554 FDA approved drugs and subjected them through a series of stringent filtering criteria of multi-level docking which resulted in 17 prioritized drugs as potential candidates for repurposing for the treatment of TB.

## Conclusion

Our study has several intrinsic advantages. Since our starting materials for virtual screening were already FDA approved drugs for other clinical use (but not for TB) and human safety issues are well established, if these drugs are proved to show efficacy for TB in vivo in animal models in future, they can be expedited for clinical research directly in TB patients. Further, drugs have been screened against one of the essential proteins, murE, which is involved in the making of cell wall of the M. tuberculosis; thus, drug-inhibitors identified for this enzyme would potentially have fatal consequence on the bacteria. The bioinformatics protocol we applied in this study has helped us to prioritize 17 drugs including those which were already reported to have in vitro activity against M. tuberculosis; this observation serves as a validation and proof-of-concept for our protocol that we have conceptualised and applied in this study. Encouraged by these results, we have undertaken some of the prioritized drugs for further experimental studies towards repurposing for tuberculosis.

## Figures and Tables

**Table 1:** Prioritized drugs which show strong binding interactions with murE of M. tuberculosis

DrugBank ID	Drug name	Primary use*	Glide score	Glide Rank	AutoDockVina Binding Affinity	ADV Rank	RMSD (Ao)	Amino acids of murE interacting with drugs
DB08995	Diosmin	Venous disease	-14.605109	1	-14.9	32	0.923	S84, T85, Q70, H91, R128, T201
DB01249	Iodixanol	Contrast agent during coronary angiography.	-12.669883	2	-17.5	20	0.924	S84, R230, E198, L67, R128, A193, T201, R68, K396, D250
DB00256	Lymecycline	Various bacterial infections	-12.614792	3	-24.3	1	0.922	S84, E198, R230, T86, R128, H248, D250
DB00284	Acarbose	Type 2 diabetes.	-12.064307	4	-24.1	2	0.91	S84, S222, R230, T195, T85, T86, Q70, L67, A79, R128, H224
DB08874	Fidaxomicin	Clostridium difficile- associated diarrhea.	-11.250336	5	-19.8	12	0.985	R424, T82
DB00224	Indinavir	HIV/AIDS	-11.217933	6	-15.3	30	0.918	S84, T195, R230, T85, T86, R128
DB00035	Desmopressin	Diabetes insipidus	-11.019141	7	-23.3	3	0.927	S84, R230, T195, T86, T85, L67, A69, Q70, R128, H248, K157
DB06663	Pasireotide	Cushing’s disease.	-10.852191	8	-18.6	17	0.975	T86, Q70
DB06810	Plicamycin	Antineoplastic antibiotic.	-10.471306	9	-19	15	0.926	S84, R230, S222, T195, T85, T86, Q70, L67, A79, K157, R128, H224
DB02638	Terlipressin	Hypotension.	-10.34453	10	-11.1	53	0.981	R230, S222, T195, T82, R424
DB00520	Caspofungin	Antifungal drug	-10.004357	12	-20.4	9	0.907	A69, K157
DB00512	Vancomycin	Staphylococci infections.	-9.474767	19	-23.2	4	0.907	T85, T86, Q70, L67
DB00407	Ardeparin	Postoperative venous thrombosis.	-7.873831	29	-20.4	10	0.911	S84, R230, S222, T195 T85, T86, Q70, L67, R128, H224
DB01141	Micafungin	Antifungal drug	-5.873	47	-23.1	5	0.901	T86
DB00290	Bleomycin	Antineoplastic, especially for solid tumors.	-3.831	48	-20.7	8	0.92	G70
DB00403	Ceruletide	Paralytic ileus.	-3.7831	50	-21.7	6	0.9	T82
DB00781	Polymyxin B Sulfate	Infections of the urinary tract, meninges, and blood stream.	-1.81	52	-20.8	7	0.97	T85
								
Amino acids in boldface are from the active site of the murE as provided in the crustal structure [11,12]. *The primary use of all of the drugs provided in this table were referred from DrugBank [14]. RMSD: Root-mean-square deviation. ADV: AutoDock Vina
